# The utility of IMP3 immunohistochemical staining in differentiating nodular lymphocyte predominant Hodgkin Lymphoma from T-Cell/Histiocyte-Rich large B-Cell lymphoma

**DOI:** 10.1186/s12885-022-10321-z

**Published:** 2022-12-28

**Authors:** Farid Kosari, Trifeh Bakhshi, Fereshteh Ameli, Maral Mokhtari

**Affiliations:** 1 Fellowship of Hematopathology, Department of Pathology, Shariati Hospital, Tehran University of Medical Science, Tehran, Iran; 2 Department of Pathology, Cancer Institute Imam Khomeini Hospital Complex, Tehran University of Medical Science, Tehran, Iran; 3 Fellowship of Hematopathology, Shiraz University of Medical Science, Shiraz, Iran

**Keywords:** Nodular lymphocyte predominant Hodgkin lymphoma, T cell/histiocyte-rich large B-cell lymphoma, IMP3, Immunohistochemistry

## Abstract

**Introduction:**

Nodular lymphocyte predominant Hodgkin lymphoma (NLPHL) and T cell/histiocyte-rich large B-cell lymphoma (THRLBCL) have overlapping histological features that make their diagnosis challenging. Insulin-like growth factor II mRNA-binding protein 3 (IMP3) is a recently proposed diagnostic marker for Hodgkin’s lymphoma. The aim of this study was to determine the ability of IMP3 in differentiating NLPHL from THRLBCL.

**Methods:**

In this retrospective study, the formalin-fixed paraffin-embedded blocks from 56 patients (28 NLPHL and 28 large B cell lymphoma (LBCL, including 16 THRLBCL and 12 DLBCL, NOS) cases based on immunohistochemistry (IHC) were included. Sample sections were stained for IMP3 using IHC method. Moderate to strong staining in at least 10% of tumor cells was considered positive IMP3 expression.

**Results:**

The mean age of the patients was 41.25 ± 16.08 years old. The majority of the patients were male. There was a significant age difference between NLPHL (34.61 ± 16.44 years old) and LBCL (47.89 ± 12.85 years) groups (*p* = 0.001). No significant difference was seen in gender and site between NLPHL and LBCL groups. The expression of IMP3 was mainly strong in LBCL group, while it was heterogeneously distributed among NLPHL samples ranging from weak to strong (*p* < 0.001). It was determined that strong IMP3 expression at 55.00% can differentiate LBCL from NLPHL with 71.4% sensitivity and 71.4% specificity.

**Conclusion:**

Our findings showed that IMP3 may be a good complement in differentiating NLPHL cases from THRLBCL.

## Introduction

Nodular lymphocyte-predominant Hodgkin lymphoma (NLPHL) is a rare lymphoma entity. NLPHL has distinct pathologic and clinical characteristics. The most common NLPHL clinical course is indolent and the disease is diagnosed at early stages in most patients. First-line treatment in of early stage (stage IA) NLPHL is limited-field radiotherapy, while combined-modality treatment is prescribed to intermediate-stage NLPHL, and chemotherapy is the only treatment in advanced NLPHL [1].

T cell histiocyte rich large B cell lymphoma (THRLBCL) is also a rare and aggressive subtype of diffuse large B cell lymphoma (DLBCL). The prognosis of THRLBCL is comparable to DLBCL, not otherwise specified (NOS). THRLBCL require more intensive chemotherapy modalities compared to NLPHL [2].

The histological features of NLPHL neoplastic cells, also called lymphocyte predominant (LP), include scant cytoplasm, a folded or multilobated nucleus, which is mostly basophilic. These neoplasms are usually CD45, CD20, CD79a, and Bcl-6 positive but do not express CD15, and are often CD30-negative [3].

Large cells in THRLBCL may look similar to LP and HRS cells; however, pleomorphism is usually more pronounced in large cells. Although the immunoprofile of large cells much resembles the LP and HRS cells, large cells less commonly express Bcl-6 and more frequently express IRF4/MUM1 compared to LP and HRS cells. Therefore, differentiating these cells is nearly impossible solely based on neoplastic cell morphology [2].

The common characteristic of NLPHL is nodular predominant background reactive B-cells predominating based on CD23 or CD35 expression in follicular dendritic cell meshwork. In contrast, THRLBCL is mainly composed of lymphoid T-cells in a diffuse background. Therefore, THRLBCL can be diagnosed based on the scattered presence of neoplastic cells in a background mainly composed of T-cell and histiocytes while lacking small B-cells [4–8].

Some less common variants of NLPHL, including pattern E, are usually diagnosed in advanced stages (stage IIB and higher) [9]. The morphologic similarities between pattern E NLPHL(diffuse, THRLBCL-like) and THRLBCL has made their diagnosis challenging [9]. Furthermore, a common diagnostic characteristic of NLPHL is the rosette formation of PD1 + cells around neoplastic cells. However, this characteristic might be absent in nodular forms of NLPHL, including NLPHL THRLBCL-like, which makes their diagnosis even harder [5–7] .

Similarity in gene expression profile of NLPHL-THRBCL-like and de novo THRLBCL is the possible reason for the observed molecular overlap between these tumor cells [7, 10]. On the other hand, the average genome imbalance in NLPHL is higher compared to THRLBCL (10.8 and 4.7, respectively) [11]. This finding does not support the hypothesis that de novo THRLBCL is a form of NLPHL progression. However, based on the findings of recent array comparative genomic hybridization studies, THRLBCL has higher genomic aberrations compared to typical and THRLBCL-like variants of NLPHL. Therefore, these similarities in gene expression profiling between NLPHL and THRLBCL suggest a pathobiological similarity and justifies various clinical presentations of these tumors [12].

The IHC profile of LP and LBCL cells is similar; therefore, there is no distinct IHC marker has been proposed to distinguish between them. Recently, several new IHC markers, including an embryo/carcinoma marker known as insulin-like growth factor II mRNA-binding protein 3 (IMP3/KOC), have been reported for CHL [13]. IMP3 is a member of Insulin-like growth factor 2 mRNA binding protein 3 (IGF2BP3) family. The IMP3 family is important in mediating RNA stability and thus regulating cell growth and migration in the early stages of embryogenesis [14–16]. However, this oncofetal protein seems to act as a carcinogen. Overexpression of IMP3 has been reported in epithelial malignancies in bladder, liver, breast, pancreas, lung, colon, ovary, and kidney, as well as sarcomas in several soft tissues. Therefore, IMP3 has been suggested as a diagnostic marker in some epithelial malignancies [17–24]. High tissue expression of IMP3 protein was reported in 98.8% of patients with classic and other types of Hodgkin’s lymphoma [25]. Therefore, IMP3 protein was also suggested as a complementary diagnostic marker in Hodgkin’s lymphoma and also differentiating Hodgkin’s lymphoma from LBCL [13]. On the other hand, a recent study reported no difference in IMP3 expression between Hodgkin and non-Hodgkin’s lymphomas [26]. As accurate and early diagnosis and subtype identification of lymphomas is lifesaving, it is necessary to find a marker to differentiate Hodgkin’s lymphoma from LBCL. The objective of this study was to investigate IMP3 expression among NLPHL and LBCL, especially THRLBCL, and evaluate the potential of IMP3 as an IHC marker in distinguishing NLPHL from LBCL.

## Materials and methods

### Study design

This cross-sectional study was approved by the Ethics Committee of the Tehran University of Medical Sciences. The study was conducted in Imam Khomeini and Dr. Shariati Hospitals, Tehran, Iran from March 2016 to March 2020.

### Study population

Patients were identified based on searching electronic records of the Pathology Departments in the mentioned hospitals. As THRLBCL is a rare entity and neoplastic cells show almost similar immunophenotype with DLBC-NOS, patients with immunohystochemical (IHC) diagnosis of LBCL (including THRLBCL(preferably) and DLBCL,NOS) with NLPHL were identified. The formalin-fixed paraffin embedded blocks of patients with documented diagnosis of both the NLPHL and LBCL were evaluated.

The formalin-fixed paraffin embedded and hematoxylin and eosin (H&E) stained blocks were obtained from hospital archives. The H&E slides were re-evaluated and pervious IHC study markers for NLPHL, including CD30, CD15, CD20, CD45 and PAX5, and for LBCL, including CD20, CD3, BCL6, CD10 and CyclinD1, were reviewed to confirm the diagnosis based on WHO 2016 Classification of Haematolymphoid Tumors [12]. The previously IHC stained blocks were selected for monoclonal staining with IMP3.

The pathological, including histological subgroup, nodal or extra nodal location, clinical data, including remission status, and demographic data, including age and gender, were obtained from the Laboratory Information System and/or the surgical department records.

Patients were given a unique code and their information remained anonymous. Blocks with inadequate tissue for IHC and incomplete medical records were excluded from the study.

### IHC study

IHC staining was performed using monoclonal rabbit anti-human IMP3 antibody (Clone EP286, IgG isotype (manufactured using Epitomics’s RabMAb® technology under U.S. Patent Nos. 5,675,063 and 7,402,409) purified from serum and prepared in 10mM PBS, pH 7.4, with 0.2% BSA and 0.09% sodium azide). Ductal adenocarcinoma of the pancreas was used as a positive control for IMP3. The sections were deparaffinized, rehydrated and were then subjected to heat antigen retrieval technique. The manufacturer standard protocol (Master Diagnostica, Spain) was used to perform immunostaining.

### IHC staining interpretation

The IHC staining was evaluated based on semiquantitative scoring using a high magnification (400x) light microscope on the unidentified samples using a 4-tiered system. The evaluation was performed independently by two pathologists (F.A and T.B), who were both blinded to the clinicopathologic parameters and outcome of the patients. Positive cytoplasmic staining was defined as immunoreactivity in at least 10% of the tumor cells [25]. Positive stained tumor cells were then scored based on the staining intensity into weak (1+), moderate (2+), or strong (3+) [25]. Any discordance in the degree of staining between the pathologists was resolved based on the consensus between two pathologists.

### Statistical analysis

Data analysis was conducted using the statistical package for social sciences (SPSS) software version 16. The Shapiro-Wilk test was used to evaluate the normality distribution of continuous variables. Continuous variables were presented using mean and standard deviation (SD) or minimum and maximum values based on normality. Categorical variables were presented using frequency and percentage. Comparison of continuous variables between groups was performed using the independent t-test, while the chi-square test was used to compare the distribution pattern of categorical variables between groups. The receiver operating characteristic (ROC) curve analysis was performed to identify the cut-off for IMP3 expression in differentiating LBCL from NLPHL. The area under curve (AUC), 95% confidence interval (CI) for AUC, cut-off and its sensitivity and specificity were reported for the analysis. The level of statistical significance was considered as *p* < 0.05.

## Results

A total of 56 samples (19, 33.9% female and 37, 66.1% male) were evaluated. The mean age of the participants was 41.25 ± 16.08 years old. Equal number (*n* = 28) of NLPHL and LBCL samples (including 16 THRLBCL and 12 DLBCL, NOS) were included in the study. Demographic characteristics of the studied samples and their comparison between NLPHL and LBCL groups are presented in Table 1. The most common tumor sites were axillary and cervical lymph nodes (17, 31.5% each) followed by inguinal lymph node (6, 11.1%) and para-aortic lymph nodes (2, 3.7%). There was a significant age difference between NLPHL (34.61 ± 16.44 years old) and LBCL (47.89 ± 12.85 years) groups (*p* = 0.001). No significant difference in gender and site between NLPHL and LBCL groups was seen.

**Table 1:** Comparison of demographic characteristics of the study samples

Variable		Total Frequency (%)	NLPHL Frequency (%)	LBCL Frequency (%)	p
Gender	Male	37 (66.1%)	17 (45.9%)	20 (54.1%)	0.397
	Female	19 (33.9%)	11 (57.9%)	8 (42.1%)	
Site	Axillary LN	17 (31.5%)	8 (47.1%)	9 (52.9%)	0.412
	Cervical LN	17 (31.5%)	12 (70.6%)	5 (29.4%)	
	Inguinal LN	6 (11.1%)	4 (66.7%)	2 (33.3%)	
	Para-aortic LN	2 (3.7%)	1 (50.0%)	1 (50.0%)	
	Submandibular LN	1 (1.9%)	1 (100.0%)	0 (0.0%)	
	Mandibular LN	1 (1.9%)	1 (100.0%)	0 (0.0%)	
	Axillary mass	1 (1.9%)	1 (100.0%)	0 (0.0%)	
	Vertebral lesion	1 (1.9%)	0 (0.0%)	1 (100.0%)	
	Cervical and axillary LN	1 (1.9%)	0 (0.0%)	1 (100.0%)	
	Skull mass	1 (1.9%)	0 (0.0%)	1 (100.0%)	
	Sternal mass	1 (1.9%)	0 (0.0%)	1 (100.0%)	
	Abdominal mass	1 (1.9%)	0 (0.0%)	1 (100.0%)	
	Omentum	1 (1.9%)	0 (0.0%)	1 (100.0%)	
	Ankle mass	1 (1.9%)	0 (0.0%)	1 (100.0%)	
	Spleen	1 (1.9%)	0 (0.0%)	1 (100.0%)	
	Brain	1 (1.9%)	0 (0.0%)	1 (100.0%)	

*LN* Lymph node, *NLPHL* Nodular-lymphocyte predominant Hodgkin’s lymphoma, *LBCL* Large B-cell lymphoma

**Fig. 1:**
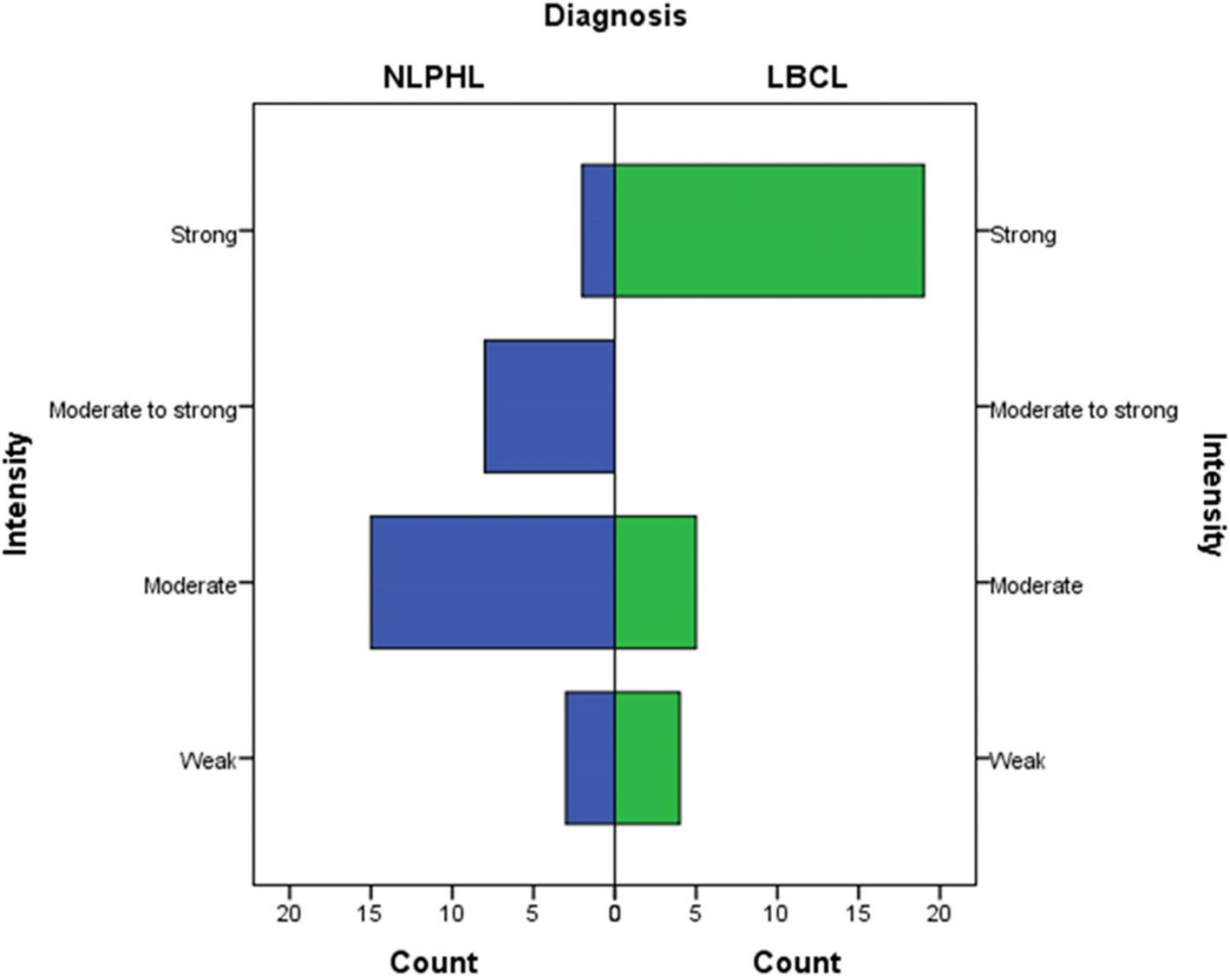
Distribution pattern of IMP3 staining intensity among NLPHL and LBCL cases

**Fig. 2:**
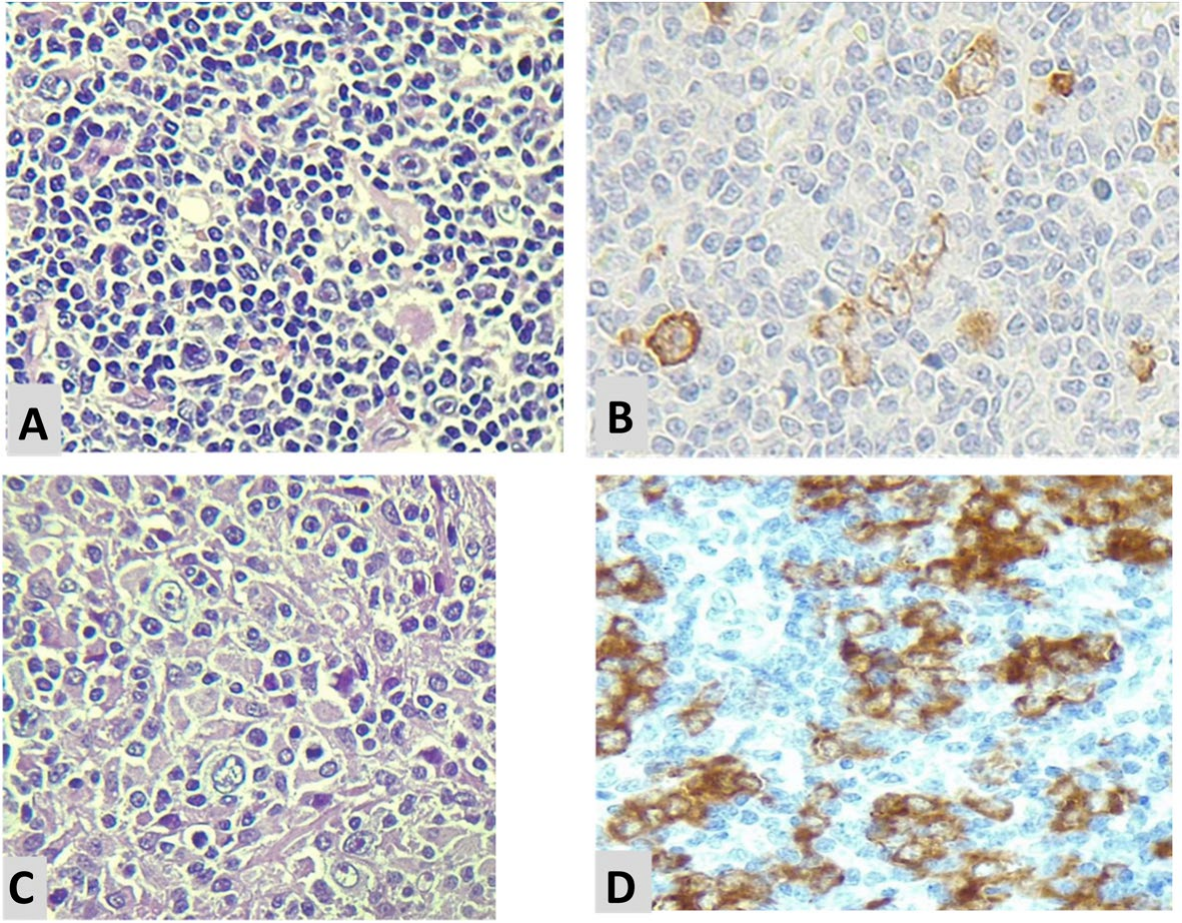
**A**) NLPHL, H&E(x400); **B**) Hetrogenous expression of IMP3 in NLPHL, IHC; **C**) THRLBCL, H&E(x400); **D**) Strong IMP3 immunoreactivity in THRLBCL

**Fig. 3:**
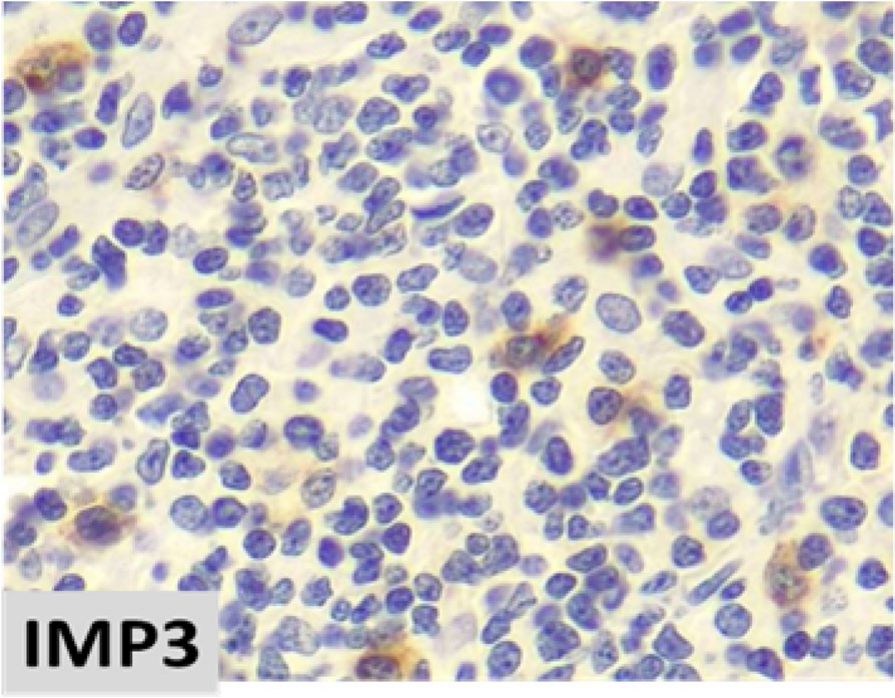
Scattered IMP3 immunreactivity in background non-neoplastic lymphoid cells in NLPHL (x400)

**Fig. 4:**
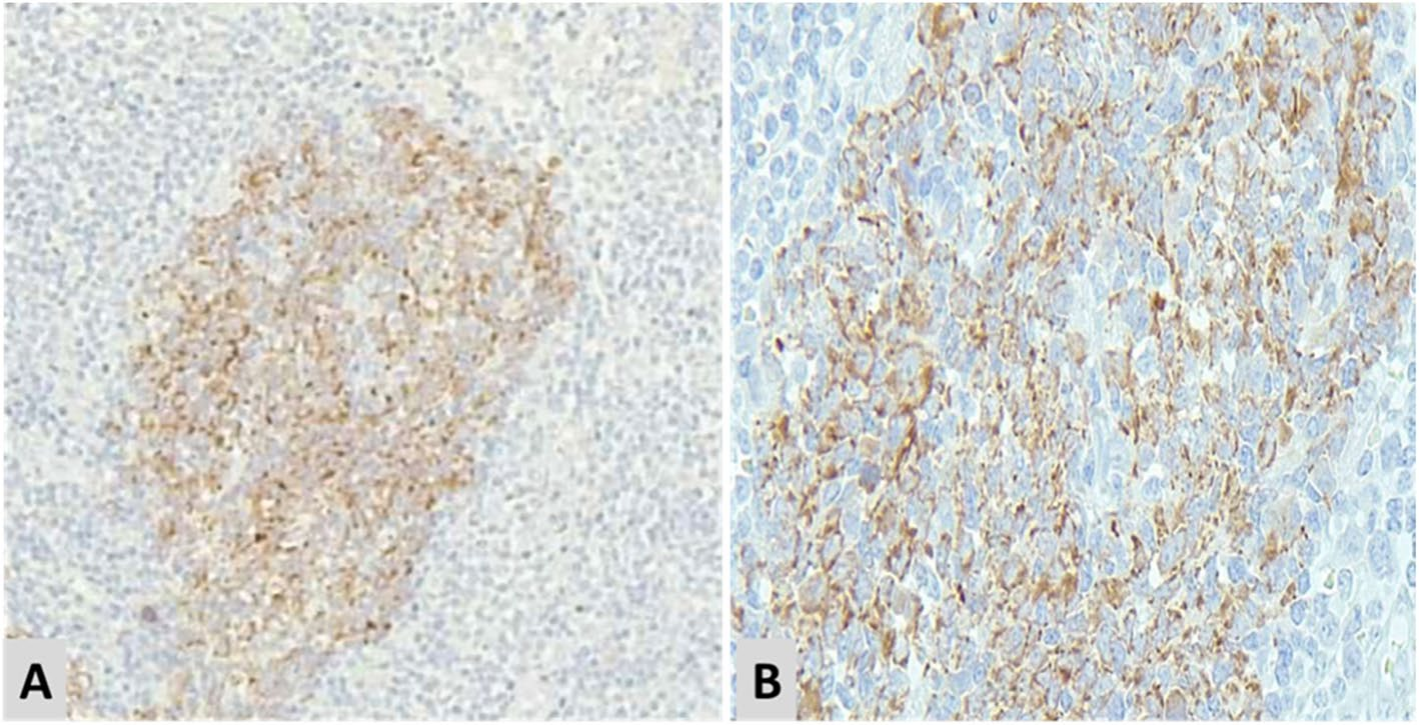
A(x100) & B(x400): IMP3 expression in reactive lymphoid germinal centers

The mean (minimum, maximum) for IMP3 expression percentage among study samples was 55.80% (5.00, 100.00). The mean (minimum, maximum) for IMP3 expression percentage among NLPHL and LBCL were 40.71 (10.00, 90.00) and 70.89 (5.00, 100.00), respectively. The IMP3 expression percentage was significantly higher in LBCL compared to NLPHL (*p* = 0.001) (Fig. 1). There was a significant difference in the distribution of IMP3 expression intensity levels between NLPHL and LBCL (*p* < 0.001). Comparison of IMP3 intensity expression in different lymphoma subtype including THRLBCL, DLBCL,NOS and NLPHL are demonstrated in Table 2. This finding indicated that the expression of IMP3 was mainly strong in LBCL group, while it was heterogeneously distributed among NLPHL samples ranging from weak to strong (Fig. 2). IMP3 was positive in in background lymphoid cells and germinal centers of reactive lymphoid follicles (Figs. 3 and 4).

**Table 2:** Comparison of IMP3 expression in different lymphoma subtype

Lymphoma type		Intensity of IMP3 expression			Total
		Negative^a^	Variable )weak to moderate)	Strong above 55%	
LBCL- subtype	THRLBCL	2	4	10	16
	DLBCL,NOS	0	1	11	12
NLPHL		3	22	3	28

^a^Moderate to strong staining in at least 10% of tumor cells was considered positive IMP3 expression and remaining was defined as negative. DLBCL NOS vs. THRLBCL *p* = 0.188; DLBCL NOS vs. NLPHL *p* < 0.001 ; THRLBCL vs. NLPHL *p* < 0.001 ; LBCL (THRLBCL + DLBCL NOS) vs NLPHL *p* < 0.001

**Fig. 5:**
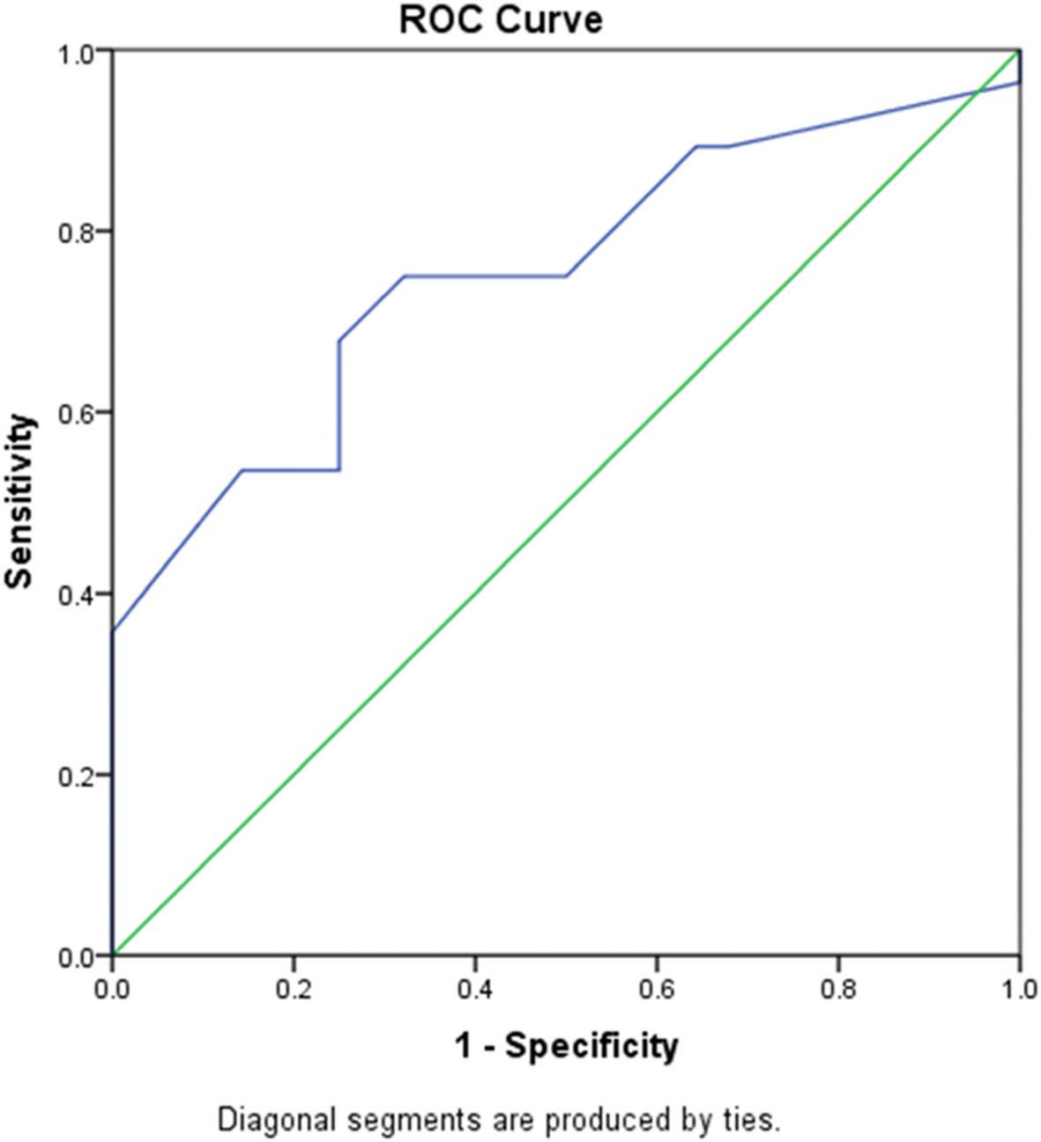
ROC curve for IMP3 expression in differentiating LBCL( including THRLBC & DLBCL,NOS) from NLPHL

Relationship between IMP3 expression and demographic variables is presented in Table 3. There was no significant relationship between IMP3 expression and demographic variables (*p* > 0.05). The ROC curve analysis was used to identify whether IMP3 expression could differentiate NLPHL from LBCL. The area under the curve for ROC curve was 75.4 (95% CI: 0.624 and 0.883) indicating that the IMP3 expression intensity is capable of differentiating these two neoplasms. It was determined that IMP3 expression at 55.00% can differentiate LBCL from NLPHL with 71.4% sensitivity and 71.4% specificity (Fig. 5). This finding indicated that IMP3 expression higher than 55% could differentiate 71.4% of LBCL tumors from NLPHL.

**Table 3:** Relationship between IMP3 expression and demographic variables

Variable	Unstandardized beta	Standardized beta	p
Age	0.320	0.159	0.574
Gender	-6.320	-0.111	0.732
Site	4.370	0.120	0.700

**Fig. 6:**
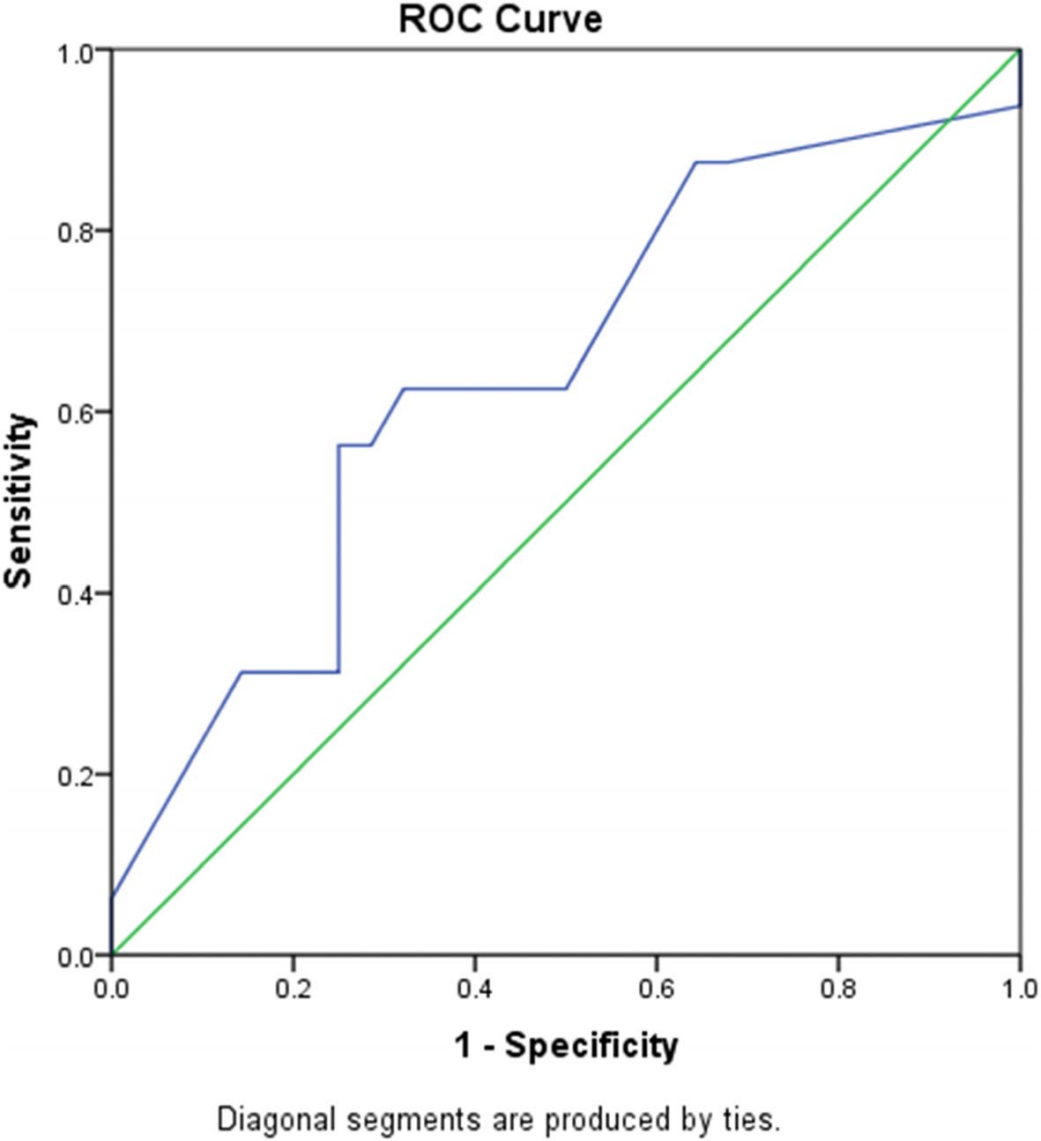
ROC curve for IMP3 expression in differentiating THRLBCL from NLPHL

Furthermore, the ROC was also performed to identify whether IMP3 expression could differentiate THRLBCL type from NLPHL. The area under curve for ROC curve was 64.3% (95% CI: 46.8% and 81.7%) indicating that IMP3 expression intensity is capable of differentiating these two neoplasms. It was determined that IMP3 expression at 55% can differentiate THRLBCL type from NLPHL with 56.3% sensitivity and 71.4% specificity (Fig. 6). This finding indicated that IMP3 expression higher than 55% could differentiate 56.3% of THRLBCL type from NLPHL.

**Fig. 7:**
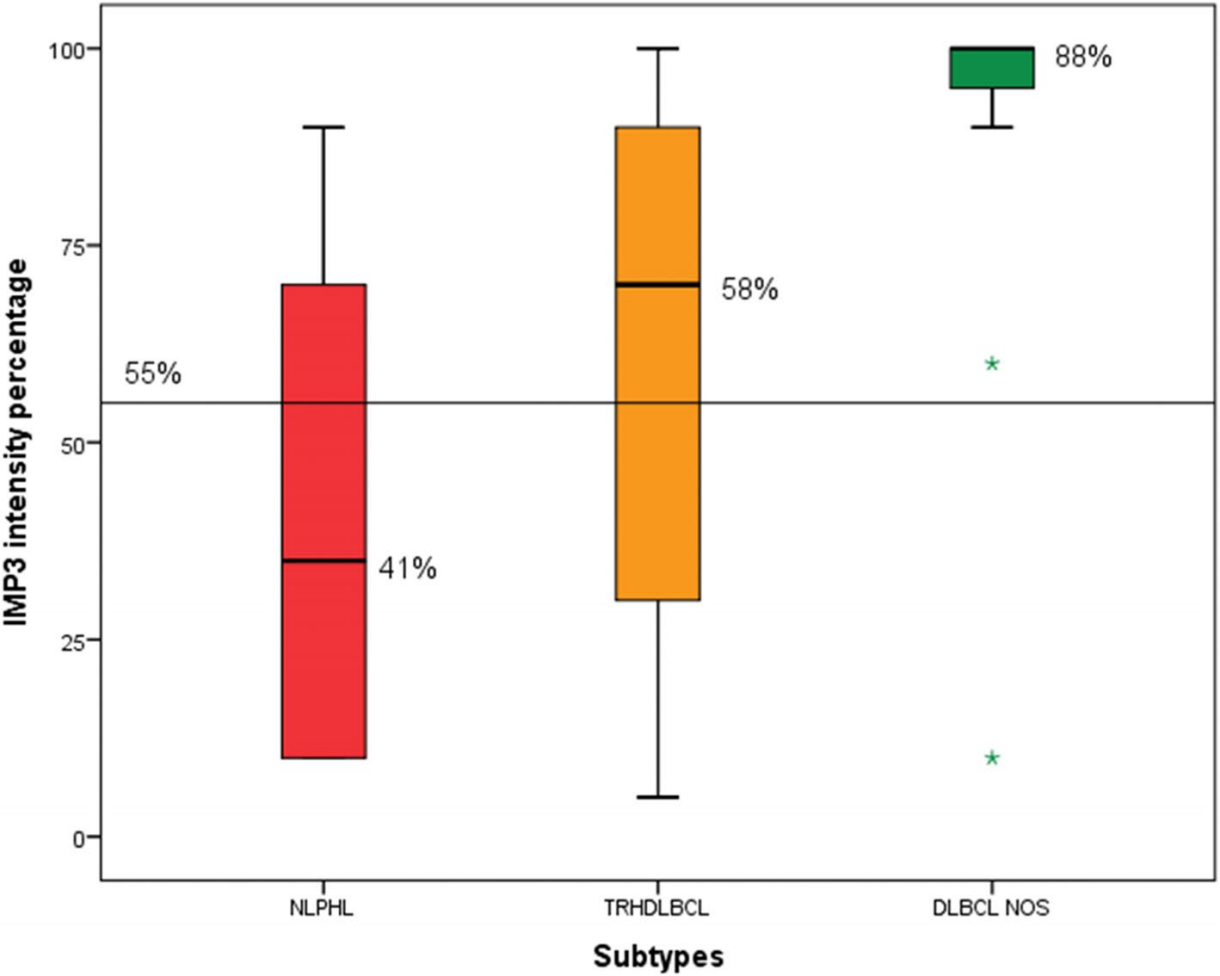
Box plot for mean, standard deviation, and range of the intensity percentage of IMP3 among NLPHL, THRLBCL and DLBCL,NOS subtypes with respect to 55% cut-off

Based on the identified cut-off for differentiating NLPHL from THRLBCL and DLBCL NOS types, the box plot indicating mean, standard deviation and range of the data with respect to 55% cut-off are presented in Fig. 7. Discussion.

The common pathological feature of both the NLPHL and THRLBCL is the presence of scattered large neoplastic B-cells in a background of benign lymphocytes and macrophages. NLPHL, especially THRLBC-like variant, and de novo THRLBCL may show significant morphologic and immunophenotypic overlap that makes the diagnostic of these two neoplasms challenging [2]. Differences in the prognosis and treatment of NLPHL and THRLBCL make their differentiation critical. The overall prognosis of NLPHL is favorable, except in advanced stages. It was reported that NLPHL has 6.3% risk of transformation to aggressive B-cell lymphoma in 10 years, which is associated with increased risk of mortality. In contrast, THRLBCL, has an aggressive nature. The disease is often refractory to the chemotherapy regimens currently in use [2, 12, 27].

Members of IMP family, including IMP1, IMP2 and IMP3, have an important role in primary embryogenesis, RNA trafficking, stabilization and regulation of the proliferation and migration of embryonic cells [27]. Not all normal tissues express IMP3. In lymphoid tissue, IMP3 is only expressed in lymph nodes, spleen and tonsillar germinal centers [28]. IMP3 expression was also reported in centrocytes, centroblasts, and thymocytes, while IMP3 is not expressed in bone marrow cells [29].

IMP3 was suggested as a diagnostic marker in differentiating Hodgkin lymphoma subtypes, including NLPHL from LBCL [13]. Our study results demonstrated that IMP3 was highly positive in both NLPHL (25/28, 89%) and LBCL (26/28, 92%). In contrast to the findings of our study, a previous study reported no IMP3 expression in DLBCL cases (0 of 90 cases) [13]. However, the findings of our study and the mentioned study were similar in terms of IMP3 positivity in NLPHL cases (25/28, 89% in our study and 12/13, 92.3% in the previous study [13]. Therefore, in our study using the proposed 10% cut off for moderate or strong staining of IMP3, could not be used as differentiation marker for NLPHL and DLBCL.

The mean (minimum, maximum) for IMP3 expression percentage among neoplastic cells in NLPHL and DLBCL were 40.71 (10.00, 90.00) and 70.89 (5.00, 100.00), respectively, which was significantly higher in LBCL compared to NLPHL (*p* = 0.001).

There was a significant difference in the distribution of IMP3 expression intensity levels between NLPHL and LBCL (*p* < 0.001). Majority of LBCL cases including both THRLBCL and DLBCL,NOS show strong expression of IMP3 with high percentage whereas LP cells in NLPHL revealed more heterogeneous and variable staining with mean expression percentage below 50%. This finding was in contrast with the findings of Mehrjerdi et al. and Rebbeca et al. studies which showed high staining intensity (3+) in 62.5% of the HL group and 21.1% in the non-Hodgkin lymphoma(NHL) with variable staining in DLBCL cases [26, 30]. A reason for this difference might be related to the inclusion of mainly classic type of Hodgkin’s lymphoma and failure to report the percentage and intensity of staining in NLPHL in the mentioned studies. Furthermore, Tang et al. reported that IMP3 was expressed in 100% (10/10) of the NLPHL cases with strong intensity in majority of cases (7/10, 70%) [23]. Another reason for the controversies in the findings of the mentioned studies might be related to the use of different clones of antibody and techniques in the studies.

Our findings indicated that strong IMP3 expression higher than 55% could differentiate 71.4% of DLBCL tumors from NLPHL. However, this sensitivity was decreased to 56.3% for THRLBCL cases and in the other words, the identified cut-off would better differentiate DLBCL NOS subtype from NLPHL, compare with THRLBCL. Further studies are required to determine whether IMP3 intensity and percentage can differentiate LBCL from NLPHL.

As neoplastic cells in NLPHL are mainly negative for CD30, IMP3 can be used to identify majority of neoplastic LP cells and could be used as complementary marker for the diagnosis of NLPHL.

In our study, IMP3 was expressed in the residual germinal centers of non-neoplastic lymphoid follicles of lymphoma cases, while other parts of lymphoid follicles were negative. This result was comparable to the results of a previous study which showed IMP3 expression in reactive lymphoid follicles (germinal center) and in germinal cell B-cell derived neoplasms (Burkitt and follicular lymphoma). On the other hand, 8–20% of the lymphomas with the origin of non-germinal center (marginal zone, mantle cell, small lymphocytic, B lymphoblastic and anaplastic large cell lymphoma) were positive for IMP3 [23]. This result could be further investigated for classification of DLBCL as germinal center or activated B cell origin.

Expression of IMP3 in background lymphoid cells, also could indicate the possibility of IMP3 expression in activated B and T cells. However, this finding should be investigated in supplementary studies.

## Conclusion

Variable staining of IMP3 in NLPHL neoplastic cells in contrast to the strong staining intensity and higher expression percentage of IMP3 in LBCL can be considered as a pathognomonic feature of THRLBCL. However, this hypothesis should be further evaluated. Furthermore, the mechanistic role of IMP3 in initiation and development of Hodgkin’s lymphoma and its potency to be used as a prognostic or therapeutic marker needs to be investigated.

## Data Availability

The data that support the findings of this study are available on request from the corresponding author (Fereshteh Ameli/TUMS) upon reasonable request. The data are not publicly available due to university regulations.
